# Audit on Decisions Relating to Cardiopulmonary Resuscitation (CPR) in 2 Older Adult Inpatient Wards

**DOI:** 10.1192/bjo.2024.617

**Published:** 2024-08-01

**Authors:** Joel Philip, Vinu Cherian, Jennifer Ford

**Affiliations:** Essex Partnership University NHS Foundation Trust, Thurrock, United Kingdom

## Abstract

**Aims:**

This audit aimed to understand the practice of advance care planning with regard to cardiopulmonary resuscitation (CPR) in an older adult inpatient setting and to ultimately improve practices to conform to nationally set standards.

The aims of the audit were:

(1) To determine the prevalence of advance care planning decisions relating to CPR in patients admitted to two older adult psychiatric wards at Thurrock Community Hospital.
(2) To confirm that the practice of discussion and documentation of ‘Do Not Attempt Cardiopulmonary Resuscitation' (DNA-CPR) decisions is consistent with current national standards.

**Methods:**

First, we scrutinized whether the patient's preferences for CPR as a life-sustaining treatment were documented or known to the primary care physician at the time of admission, and whether there was a DNA-CPR order in place at the time of admission.

Next, we looked at whether a discussion about CPR was facilitated with the patient (or those close to the patient) during the admission, whether the patient was involved in the discussion surrounding CPR and the reasons for their exclusion (if excluded), and at what point in time during the admission this discussion was carried out and whether it was properly documented.

Finally, we assessed the level of completion of the DNA-CPR form itself.

Total sample: 38 patients.

**Results:**

13 out of 38 patients (34.21%) had a DNA-CPR form in place.

10 out of 13 DNA-CPR forms (76.92%) were complete in all aspects.

Discussion relating to DNA-CPR was not carried out in 29 out of 38 patients (76.32%) during their current admission.

Mental Capacity Assessments and Best Interest meetings were not documented as having been carried out as was necessary in the 4 patients (0%) who did not have a designated Lasting Power of Attorney.

**Conclusion:**

Discussions about advance care planning and DNA-CPR were not being carried out in a timely manner as per the national guidelines.

